# The Critical Modulatory Role of Spiny Stellate Cells in Seizure Onset Based on Dynamic Analysis of a Neural Mass Model

**DOI:** 10.3389/fnins.2021.743720

**Published:** 2021-12-24

**Authors:** Saba Tabatabaee, Fariba Bahrami, Mahyar Janahmadi

**Affiliations:** ^1^Human Motor Control and Computational Neuroscience Laboratory, School of Electrical and Computer Engineering (ECE), College of Engineering, University of Tehran, Tehran, Iran; ^2^Department of Physiology, Neuroscience Research Center, School of Medicine, Shahid Beheshti University of Medical Sciences, Tehran, Iran

**Keywords:** neural mass model, bifurcation analysis, linear/non-linear resonance, excitatory interneurons, preictal state

## Abstract

Growing evidence suggests that excitatory neurons in the brain play a significant role in seizure generation. Nonetheless, spiny stellate cells are cortical excitatory non-pyramidal neurons in the brain, whose basic role in seizure occurrence is not well understood. In the present research, we study the critical role of spiny stellate cells or the excitatory interneurons (EI), for the first time, in epileptic seizure generation using an extended neural mass model inspired by a thalamocortical model originally introduced by another research group. Applying bifurcation analysis on this modified model, we investigated the rich dynamics corresponding to the epileptic seizure onset and transition between interictal and ictal states caused by EI connectivity to other cell types. Our results indicate that the transition between interictal and ictal states (preictal signal) corresponds to a supercritical Hopf bifurcation, and thus, the extended model suggests that before seizure onset, the amplitude and frequency of neural activities gradually increase. Moreover, we showed that (1) the altered function of GABAergic and glutamatergic receptors of EI can cause seizure, and (2) the pathway between the thalamic relay nucleus and EI facilitates the transition from interictal to ictal activity by decreasing the preictal period. Thereafter, we considered both sensory and cortical periodic inputs to study model responses to various harmonic stimulations. Bifurcation analysis of the model, in this case, suggests that the initial state of the model might be the main cause for the transition between interictal and ictal states as the stimulus frequency changes. The extended thalamocortical model shows also that the amplitude jump phenomenon and non-linear resonance behavior result from the preictal state of the modified model. These results can be considered as a step forward to a deeper understanding of the mechanisms underlying the transition from normal activities to epileptic activities.

## Introduction

Epilepsy is one of the most common disorders of the central nervous system (CNS), which causes sudden abnormal and synchronized brain activities, resulting in seizures. Interactions between excitatory and inhibitory neurons shape mainly brain activities, and some transitory disparity in the inhibitory/excitatory balance can trigger a seizure. One-third of people with epilepsy are likely to have drug-resistant epilepsy, and treatments such as surgery or stimulation-based treatments like deep brain stimulation (DBS) can be considered for these kinds of drug-resistant patients (Shan et al., 2021). A seizure can be composed of four distinct states including preictal, ictal, interictal, and postictal. The preictal state appears before the seizure begins and indicates that seizures do not simply start out of nothing. Experimental studies (Bartolomei et al., 2004; Huberfeld et al., 2011) on human tissue and intracranial EEG signals have shown that preictal spikes are distinguishable from ictal and interictal signals. The preictal signal can be used to predict seizure occurrence. Understanding the mechanism of generation of the preictal period opens the way for a more precise seizure prediction and thereby more reliable automatic interventions to prevent the seizure occurrence (Moghim and Corne, 2014).

Different seizures can be categorized into focal and generalized onset seizures. Tonic, clonic, and absence seizures are the generalized onset types of seizures, and they occur when a widespread activity triggers in both hemispheres of the brain (Scheffer et al., 2017). Furthermore, there are two groups of seizures that have known hemispheric origins, i.e., generalized and focal onset seizures (Fisher, 2017). Both groups are categorized into two subclasses: motor and non-motor seizures. Both subclasses have several motor seizure types in common (e.g., tonic and clonic seizures). However, each type has different manifestations and symptoms. On the other hand, absence seizures are among well-known non-motor generalized onset seizure groups that are also considered as sensory and emotional seizures (Fisher, 2017). In tonic–clonic seizures, a clonic activity follows the tonic activity. Electrophysiological observations have shown the two-way transition between absence and tonic–clonic seizures (Mayville et al., 2000), and dysfunction of the cortical and/or thalamic circuitries is believed to produce the absence, clonic, and tonic epileptic activities, together with transitions between them. Recorded electroencephalogram (EEG) signals indicate that seizures can vary in frequency contents. A typical absence seizure can be considered as approximately synchronous spike and wave discharges (SWD) with a frequency of 2∼4 Hz, but atypical absence seizures (another kind of non-motor generalized onset seizures) show different frequency ranges (<2 and >4 Hz) (Velazquez et al., 2007). A tonic seizure is a fast-spiking activity (>14 Hz) with low amplitude, and a clonic seizure is slow-wave activity (approximately 3 Hz) with high amplitude.

Several studies have shown that interneurons are cell types, which play key roles in the initiation and termination of epileptic seizures (Khan et al., 2018; Tran et al., 2020). Interneurons in the cortex not only control the activity of pyramidal neurons but also receive thalamic relay nucleus inputs. Therefore, they are important factors in transferring and integrating the sensory information coming from the thalamus to the cortex (Danober et al., 1998). Interneurons are traditionally regarded as inhibitory neurons, but more precisely, there are two kinds of interneurons in the CNS, i.e., excitatory and inhibitory interneurons. Inhibitory interneurons use the neurotransmitter gamma-aminobutyric acid (GABA) or glycine, and excitatory interneurons (EI) are spiny stellate cells in the neocortex of the human brain and use glutamate as their neurotransmitters (Okhotin, 2006). A study on the human brain (Steriade and Contreras, 1998) has shown that the neurons in the neocortex play a crucial role in SWD. Another study on the juvenile mice using multi-photon imaging (Neubauer et al., 2014) suggested that the neocortex has an intrinsic predisposition for seizure generation and pathological recruitment of the thalamus into joint synchronous epileptic activities. A genetic mutation study on mice has shown that in the spiny stellate cells (in the neocortex), an alteration in the kinetic of N-methyl-D-aspartate receptors (NMDA) was sufficient to cause neuronal hyper-excitability leading to epileptic activity in the brain (Lozovaya et al., 2014). An experimental study on monkeys has shown that synchronous discharges of EI could spread the epileptic activity in the brain, and EI could play an important role in the initiation and propagation of SWD, in which the spike is because of the extracellularly synchronous and powerful depolarization of EI, and the wave is because of the inhibitory interneurons. Therefore, SWD can be generated as a result of the interactions between inhibitory and EI (Steriade, 1974). Nevertheless, it is not well understood how EI in the neocortex of the human brain can explicitly cause a seizure. Therefore, understanding the epileptogenic role of the EI in the neocortex of the human brain is crucial to deepen our knowledge about neocortical epilepsy.

On the other hand, computational modeling of epilepsy has provided dynamic insights into the mechanism underlying the transition from normal to epileptic activities. Fan et al. (2015) used a modified computational field model of a cortical microzone and showed that the two-way transitions between absence and tonic–clonic epileptic seizures are induced by disinhibition between slow and fast inhibitory interneurons. Nevertheless, they ignored the role of thalamic circuitry and its interaction with the cerebral cortex in the proposed computational model, whereas, in Zhang et al. (2015) and Law et al. (2018) it is shown that the mechanism of seizures depends on the dysfunctionality in the function of the thalamus and or cortex. Experimental evidence suggests that interactions between the thalamus and cerebral cortex influence the initiation and propagation of SWD (Pinault and O’Brien, 2005). For that reason, Taylor et al. (2014) developed a thalamocortical model to investigate the SWD generation. Liu and Wang (2017) introduced a thalamocortical model inspired by the model developed by Taylor et al. (2014) and Fan et al. (2015). In their model, they considered the dual pathways between fast and slow inhibitory interneurons in the cortex and simulated normal activity, clonic, absence, and tonic seizures. However, none of the models mentioned above did consider the EI population in the thalamocortical circuitry, and therefore, they did not study the role of the EI population in transition from the interictal to the ictal state.

In the present study, we modified and extended a thalamocortical model, originally proposed by Liu and Wang (2017). The original model describes absence, tonic, clonic, and tonic–clonic seizure generation. That is one of the reasons we chose this model and extended it to investigate the role of EI on seizure onset using dynamical analysis. For this purpose, we introduced an additional group of interneurons into the model, which was excitatory and considered their interactions with pyramidal neurons, inhibitory interneurons, and thalamic relay nucleus in the brain. As will be shown, this model not only is capable of producing different types of epileptic seizures such as absence, clonic and tonic, but also generates the preictal period. The original model of Liu and Wang (2017) does not describe the preictal period.

The organization of the paper is as follows. In section “Materials and Methods,” the modified epileptic dynamical model inspired by the model proposed in Liu and Wang (2017) is introduced. Then, in section “Results,” we first explore the role of impairment in the activity of the EI in interictal to ictal transition. Using the modified model, we investigate the role of GABAergic and glutamatergic receptors of EI in seizure generation and transition between interictal and ictal states. Through numerical simulations and bifurcation analysis, we show that dysfunction of excitatory and inhibitory receptors of EI leads to interictal to ictal transition. Moreover, we investigate the effect of impairments in the interactions between the thalamic relay nucleus and EI and we will investigate how they give rise to interictal to preictal transition and also facilitate the occurrence of an epileptic seizure. Then, we investigate the behavior of the extended model in a more general framework when the EI function properly, but there are dysfunctions in the thalamus, more exactly in the synaptic strength between the thalamic relay nucleus and reticular nucleus. Finally, in section “Discussion,” we examine the frequency responses of the modified thalamocortical model subjected to various sensory and cortical periodic inputs to identify the effect of various stimuli on epileptic seizures. The obtained results show that the model starts its non-linear resonator behavior when the shift from normal activity to preictal state takes place. Therefore, the amplitude jump phenomenon corresponding to chaotic behavior takes place before the onset of a seizure.

## Materials and Methods

### Model Structure

**FIGURE 1:**
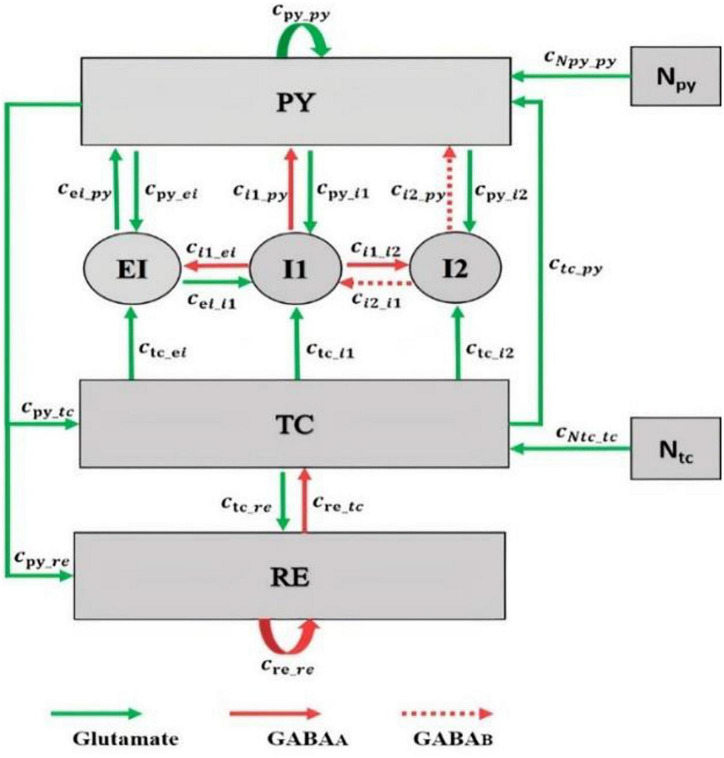
Schematic of the thalamocortical model including the excitatory interneuron population in the cortex. The model involves cortical and thalamic subnetworks. In the cortical subnetwork, PY is the pyramidal neuronal population, EI is the excitatory interneurons population, and I1 and I2 are the fast and slow inhibitory interneurons populations, respectively. In the thalamic subnetwork, TC is the thalamic relay nucleus population and RE is the thalamic reticular nucleus population. Green arrows define the excitatory glutamatergic receptor. Red solid and dashed arrows define the GAGA_A_ and GABA_B_ inhibitory receptors, respectively. The value of parameters of this model are as follows: c_py_py_ = 1.89, c_py_i1_ = 4, c_i1_py_ = 1.8, c_re_re_ = 0.01, c_tc_re_ = 10, c_re_tc_ = 1.4, c_py_tc_ = 3, c_py_re_ = 1.4, c_tc_py_ = 1, c_py_i2_ = 1.5, c_tc_i1_ = 0.05, c_tc_i2_ = 0.05, c_ei_i1_ = 0.05, c_ei_py_ = 0.442, c_i2_py_ = 0.05, c_i2_i1_ = 0.1, c_i1_i2_ = 0.5, c_Npy_py_ = 1, c_Ntc_tc_ = 1, tau_1_ = 21.5, tau_2_ = 31.5, tau_3_ = 0.1, tau_4_ = 4.5, tau_5_ = 3.8, tau_6_ = 3.9, h_py_ = –0.4, h_i1_ = –3.4, h_i2_ = –2, h_ei_ = –1, h_tc_ = –2.5, h_re_ = –3.2, ε = 250,000, a_tc_ = 0.02, a_py_ = 0.02, B_Npy_ = 0.7, and B_Ntc_ = 0.1. These parameters were obtained by trials and error such that the dynamic behavior of the model (i.e., the bifurcation diagrams) was the same as the original model developed by Liu and Wang (2017).

Recent studies Jiang et al. (2018) and Zhang et al. (2020) have shown that during generalized seizures, dysfunctionality has been identified in the thalamocortical network. Therefore, both the cortex and thalamus play an important role in seizure generation. Accordingly, we modified the thalamocortical model of Liu and Wang (2017) and as shown in Figure 1. This model is a neural mass model, which considers the inhibitory and excitatory neuronal populations and their connections in the brain. The cortical section of this model includes excitatory pyramidal neuron population (PY) and mutual fast and slow inhibitory interneuron populations (I1, I2) having inhibitory GABA_A_ and GABA_B_ receptors. Activation of ionotropic GABAA receptors causes fast inhibitory postsynaptic potentials (IPSPs) by allowing the influx of Cl^–^ into the postsynaptic cells, while activation of metabotropic GABAB receptors mediates a slow inhibition by inducing K^+^ efflux. The thalamic subsystem includes a population of excitatory thalamic relay nucleus (TC) and the inhibitory population of neurons located in the reticular nucleus (RE). To investigate the effect of spiny stellate cells on seizure onset, we considered the EI population, EI, in our thalamocortical model (see Figure 1). In this new model version, we will investigate the synaptic connectivity strength of EI to explore dynamics, which lead to preictal state and dynamics during transitions between absence, tonic, and clonic seizures.

In this thalamocortical model, we analyze the interactions between neuronal populations in the network by varying the strength of the synaptic connections. These synaptic connections are associated with the type of receptors. In the brain, receptors are either excitatory (glutamatergic) or inhibitory (GABAergic). In the cortex, the pyramidal population is excitatory n and there is a mutual excitatory connection between pyramidal and EI by the glutamatergic receptors (Feldmeyer et al., 2002). Excitatory and inhibitory interneurons are locally (Sun et al., 2006), and based on this fact, we consider an excitatory and inhibitory connection between EI and fast inhibitory interneurons. The thalamus is globally connected to the EI by the thalamic relay nucleus, and the thalamic relay nucleus is regarded as an excitatory population (da Costa and Martin, 2011). Here, we consider an excitatory connection from the thalamic relay nucleus population to the EI.

The thalamic relay nucleus receives all the sensory information from different parts of the brain, and then this information is sent to the appropriate area in the cortex for further processing (Taylor et al., 2015; Castejon et al., 2016). Here, we investigate the dynamic of the extended model when it receives cortical and sensory inputs. In this regard, we consider an input (N_tc_) to the thalamic relay nucleus by adding a periodic sensory input with frequency ftc, bias B_Ntc_, and amplitude a_tc_. Moreover, we consider a cortical input (N_py_) to the pyramidal neuronal population by adding a biased sinusoidal waveform with bias of B_Npy_, frequency of fpy, and amplitude of a_py_.

We implement the model using equations developed by Liu and Wang (2017) and the Amari neural field equations (Amari, 1977) (for the EI population). The differential equations of the model are given below.


(1) $$\frac{\text{dPY}}{\text{dt}}={\text{tau}}_{1}({\text{h}}_{\text{py}}-\text{PY}+{\text{c}}_{\text{py\_py}}f\left(\text{PY}\right)-{\text{c}}_{\mathrm{i1}\,\_\,\mathrm{py}}f\left(\mathrm{I1}\right)+{\text{c}}_{\text{tc\_py}}f\left(\text{TC}\right)-{\text{c}}_{\mathrm{i2}\,\_\,\mathrm{py}}f\left(\mathrm{I2}\right)+{\text{c}}_{\text{ei\_py}}f\left(\text{EI}\right))+\text{N}{}_{\text{py}}$$

(2) $$\frac{d\,I\,1}{d\,t}=tau_{2}(h_{i\,1}-I1+c_{p\,y\,\_\,i\,1}f\left(PY\right)-c_{i\,2\,\_\,i\,1}f\left(I2\right)+c_{t\,c\,\_\,i\,1}f\left(TC\right)+c_{e\,i\,\_\,i\,1}f\left(EI\right))$$

(3) $$\frac{d\,I\,2}{d\,t}=tau_{3}(h_{i\,2}-I2+c_{p\,y\,\_\,i\,2}f\left(PY\right)-c_{i\,1\,\_\,i\,2}f\left(I1\right)+c_{t\,c\,\_\,i\,2}f\left(TC\right))$$

(4) $$\frac{d\,E\,I}{d\,t}=tau_{4}(h_{e\,i}-EI+c_{p\,y\,\_\,e\,i}f\left(PY\right)-c_{i\,1\,\_\,e\,i}f\left(I1\right)+c_{t\,c\,\_\,e\,i}f\left(TC\right))$$

(5) $$\frac{d\,T\,C}{d\,t}=t\,a\,u_{5}\,\left(h_{t\,c}-T\,C+c_{p\,y\,\_\,t\,c}\,f\,\left(P\,Y\right)-c_{r\,e\,\_\,t\,c}\,f\,\left(R\,E\right)\right)+N_{t\,c}$$

(6) $$\frac{d\,R\,E}{d\,t}=tau_{6}(h_{r\,e}-RE+c_{p\,y\,\_\,r\,e}f\left(PY\right)-c_{r\,e\,\_\,r\,e}f\left(RE\right)+c_{t\,c\,\_\,r\,e}f\left(TC\right))$$

(7) $$N_{p\,y}=B_{N\,p\,y}+a_{p\,y}\,s\,i\,n\,\left(2\,\pi \,f\,p\,y\,t\right)$$

(8) $$N_{t\,c}=B_{N\,t\,c}+a_{t\,c}\,s\,i\,n\,\left(2\,\pi \,f\,t\,c\,t\right)$$

(9) M⁢o⁢d⁢e⁢l⁢O⁢u⁢t⁢p⁢u⁢t=(P⁢Y+I⁢1+I⁢2+E⁢I)/4

Dimensionless parameters c_1,…,16,iny,ei,in1,in2_ are the connectivity parameters, which determine the coupling strength between the populations, and h_py,i1,i2,ei,tc,re_ are input parameters, tau_1,2,…,6_ are time scale parameters, and f(x) = 1/(1 + ε^−x^) is the transfer function, where ε determines the steepness and x = PY,I1,I2,EI,TC and RE. N_py_ (cortical input) and N_tc_ (sensory input) are inputs to the pyramidal population and thalamic relay nucleus population, respectively. These parameters were obtained by trials and error such that the dynamic behavior of the model (i.e., the bifurcation diagrams) has a similar mechanism as the model developed by Liu and Wang (2017). The output of the model is taken as the mean activity of the four cortical populations.

### Simulations

Simulations are performed by the standard fourth-order Runge–Kutta integration using MATLAB 9.4 and the MatCont environments, with a step size of 0.0039 s. We set the time window at 60 s and use the last 2 s of the time series to analyze the stable state of the time domain and the deterministic behavior of the model. We use the frequency domain and time domain analyses to explore the transitions from interictal to ictal states. We extract the dominant frequency (the frequency that carries the maximum energy) from the power spectral density using the fast Fourier transform, and for the bifurcation analysis, we extract the extrema (local maximum and minimum) of the mean of four cortical populations from the time series. By doing so, we can observe seven different epileptic activities such as interictal state (or the normal background activity), preictal state, which occurs before the seizure onset, slow rhythmic activity that can be observed at seizure onset or during the seizures, typical absence seizures, atypical absence seizures, and tonic and clonic seizures. For 1D bifurcation diagrams (sections “Transition Dynamics Produced by GABAergic Receptor-Mediated Inhibition in EI,” “Transition Dynamics Produced by Glutamatergic Receptor-Mediated Excitation in Excitatory Interneurons,” and “Transition Dynamics Produced by Glutamatergic Receptor-Mediated Excitation in Reticular Nucleus”), we obtain the maximum and minimum of model output as we change the bifurcation parameter. For hybrid bifurcation analysis (section “Hybrid Cooperation of GABAergic and Glutamatergic Receptors of EI in Epileptic Transition Dynamics”) when both of the parameters of c_py_ei_ and c_i1_ei_ are changed, we computed the local maxima, local minima, and frequency of the last two seconds of the model output, and then we sorted the local maxima and minima from large to small to obtain the largest (Pmax1) and the smallest (Pmax2) maxima and the largest (Pmin1) and the smallest (Pmin2) minima. We summarized our categorization in Table 1.

**TABLE 1:** Specific characteristics of seven simulated signals.

Type of the signal	Dominant frequency range	The largest (Pmin1) and the smallest minima (Pmin2)	The largest (Pmax1) and the smallest maxima (Pmax2)
Normal background activity	DF = 0 Hz	–	–
Preictal spikes	DF < 3.5 Hz	Pmin1-Pmin2 < 0.2	0.01 < Pmax1-Pmax2 < 0.12
Slow rhythmic activity	DF < 15 Hz	–	–0.8 < Pmax1 <–0.1
Typical absence seizure	DF ∈ [2,4] Hz	0.004 < Pmin1-Pmin2	–
Atypical absence seizure	DF > 4 Hz and DF < 2 Hz	0.01 < Pmin1-Pmin2	–
Clonic seizure	DF ∈ [2,4] Hz	Pmin1-Pmin2 < 0.15	0.01 < Pmax1-Pmax2
Tonic seizure	DF ≥ 14 Hz	Pmin1-Pmin2 < 0.01	0.01 < Pmax1-Pmax2

*Dominant frequency, the largest minima, the largest maxima, the largest minima, and the largest maxima are characteristics used to separate seven different mean activities of the cortical populations.*

## Results

In this section, the role of EI in transition from interictal to ictal and the frequency response of the thalamocortical model, when it receives sensory and cortical inputs, are examined using various bifurcation analyses and dynamical simulations. In section “Transition Dynamics,” to explore the transition dynamics of the model we assume that the inputs of the model are constant, i.e., N_py_ = B_Npy_ and N_tc_ = B_Ntc_. In section “Frequency Analysis of the Different Initial States of the Model,” for investigating the frequency response of the model, a sinusoidal waveform signal is added to both sensory and cortical inputs, that is, N_py_ = B_Npy_ + a_py_sin(2*fpy*t) and N_tc_ = B_Ntc_ + a_tc_sin(2ftct).

### Transition Dynamics

EI are connected to the pyramidal neurons and fast inhibitory interneurons by glutamatergic and GABAergic receptors, respectively. The synaptic strength is not static, and the changes in the neurotransmitters released by the excitatory and inhibitory neurons in the brain can result in short-term or long-term changes in synaptic strength. Antiepileptic drugs either block glutamatergic receptors or facilitate the function of GABAergic receptors, which result in a change in the glutamatergic and GABAergic neurotransmitter release (Rogawski, 2011; Vashchinkina et al., 2014). Moreover, a ketogenic diet can bring about the altered function of receptors and change in neurotransmitter release (Zhang et al., 2018). Experimental studies have shown that changes in the function of GABAergic and glutamatergic receptors in the cortex of rats can be seen in genetic absence, tonic, and clonic seizure onset (Cortez et al., 2004; Jones et al., 2008; Errington et al., 2011). However, the transition dynamics from interictal to ictal and transitions between absence, tonic, and clonic seizures caused by the abnormalities in glutamatergic and GABAergic receptors of EI are still unclear. In this section, by using bifurcation analysis, we explore the effect of impairment in the function of GABAergic and glutamatergic receptors of the EI population on the epileptic dynamics of the model as the synaptic strengths c_i1–ei_ and c_py–ei_ change, respectively.

#### Transition Dynamics Produced by GABAergic Receptor-Mediated Inhibition in Excitatory Interneurons

**FIGURE 2:**
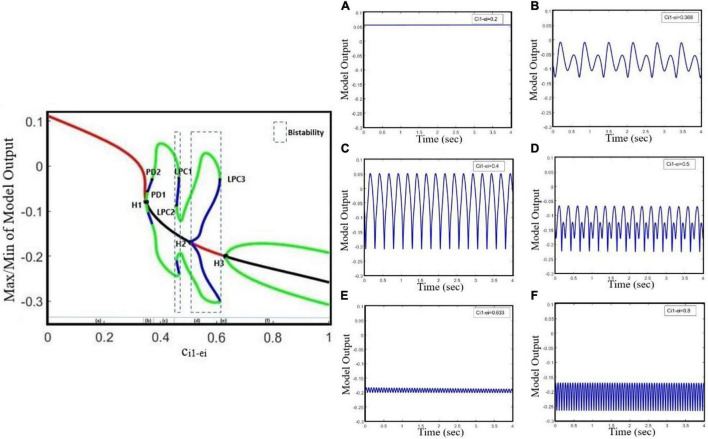
Bifurcation diagram and corresponding time series of the model output for different values of c_i1_ei_. The model output is defined as the mean value of the output voltage of PY, I1, I2, and EI populations. The bifurcation diagram of the model (left) is calculated and plotted for c_i1_ei_ as the bifurcation parameter and with c_py_ei_ = 0.8, **ctc_ei** = 4.5, a_tc_ = 0, and a_py_ = 0. We also set a_tc_ = 0 and a_py_ = 0. In the plot, blue and green cycles represent the unstable and stable limit cycles, respectively, and red and black lines represent the stable and unstable equilibrium points, respectively. According to the diagram as the parameter c_i1_ei_ changes, the model produces **(A)** normal background firing, **(B)** preictal spikes, **(C)** clonic discharges, **(D)** SWD, **(E)** slow rhythmic activity, and **(F)** tonic discharges. LPC1, LPC2, and LPC3 are fold limit cycle bifurcation, H1 and H3 are supercritical Hopf bifurcation, H2 is subcritical Hopf, and PD1 and PD2 are periods of doubling bifurcations.

Increasing the GABAergic inhibition is traditionally believed to suppress epileptic seizures; however, a computational study has shown that before seizure onset, the activity of GABAergic interneurons increases, leading to synchronous neuronal activity and epileptic seizures (Rich et al., 2020). Based on an experimental study on humans, antiepileptic drugs such as midazolam that increase the GABAergic neurotransmitters in the brain can trigger seizure (Montenegro et al., 2001). Abnormality in GABA_A_ receptors brings about a shift in the chloride reversal potential in neurons, which in turn results in changing the behavior of GABA from inhibitory to excitatory behavior and cause seizure (Khalilov et al., 2003). Therefore, the role of GABAergic receptor epileptic seizures is more complex than one could assume that they have only inhibitory roles and anticonvulsant agents always inhibit their function. Here, we investigate the effect of increasing inhibitory function of GABAergic receptors of EI in the neocortex on seizure generation. We explore the epileptiform activity induced by the dysfunction of the GABAergic receptor of the EI population as the synaptic strength c_i1–ei_ changes. Results of Figure 2 show that the model displays rich dynamics as the parameter c_i1–ei_ varies.

In Figure 2, the overall bifurcation is shown in detail for variations of c_i1–ei_ as the bifurcation parameter. In the bifurcation diagram from left to right, the red line corresponds to the stable fixed points, which are related to the normal background activity (Figure 2A), and upon increasing c_i1–ei_ to ∼0.349, a stable fixed point coalesces with a stable limit cycle and a supercritical Hopf bifurcation point (H_1_) happens; with increasing of c_i1–ei_ to ∼0.355, a period doubling bifurcation (PD1) occurs, which forms the preictal spike patterns (transition from normal background activity to ictal activity, Figure 2B). With a further increase of the c_i1–ei_ parameter to ∼0.37, another period doubling bifurcation (PD2) happens and the transition from preictal state to clonic seizure takes place and shapes clonic seizure patterns (Figure 2C). In addition, as c_i1–ei_ is increased up to ∼0.458, the first fold limit cycle bifurcation (LPC1) occurs, and another fold limit cycle bifurcation (LPC2) takes place with further increase in c_i1–ei_. With the coexistence of two stable limit cycles in the range of c_i1–ei_ bounded by twofold limit cycle bifurcations (LPC1 and LPC2), a bistable region appears and creates the coexistence of two different amplitude SWD patterns (Figure 2D). Moreover, stable points appear again at c_i1–ei_ around 0.508, where the first subcritical Hopf bifurcation (H_2_) takes place, and another bistable region turns out with coexistence of stable fixed points and a stable limit cycle until the third fold limit cycle bifurcation (LPC3) at c_i1–ei_ ∼ 0.611 takes place. Upon increasing the c_i1–ei_, the stable focus points, which are corresponding to slow rhythmic activity, take place (Figure 2E), and then at c_i1–ei_ ∼ 0.634 another supercritical Hopf bifurcation (H_3_) happens and shapes the tonic seizure patterns (Figure 2F).

#### Transition Dynamics Produced by Glutamatergic Receptor-Mediated Excitation in Excitatory Interneurons

**FIGURE 3:**
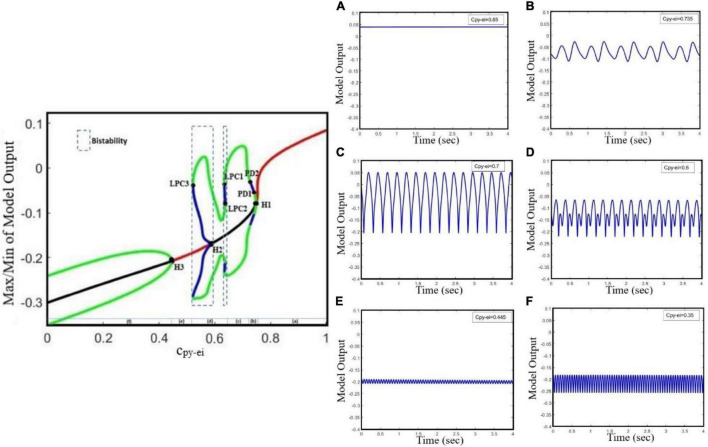
Bifurcation diagram and corresponding time series of the model output for different values of c_py–ei_. The bifurcation diagram of the model is calculated and plotted for c_py–ei_ as the bifurcation parameter with c_i1–ei_ = 0.3 and c_tc–ei_ = 4.5. We also set a_tc_ = 0 and a_py_ = 0. In the bifurcation plot, blue and green cycles represent the unstable and stable limit cycles, respectively, and red and black lines represent the stable and unstable points, respectively. It can be found from the bifurcation diagram that as the parameter c_py–ei_ decreases, **(A)** normal background firing (interictal), **(B)** preictal spikes, **(C)** clonic discharges, **(D)** SWD, and **(E)** slow rhythmic activity and tonic discharges can appear. **(F)** Tonic discharges. LPC1, LPC2, and LPC3 are fold limit cycle bifurcation, H1 and H3 are supercritical Hopf bifurcation, H2 is subcritical Hopf, and PD1 and PD2 are period-doubling bifurcations.

Studies have shown that antiepileptic drugs that are expected to reduce excitation in the brain, on the contrary, can have a paradoxical effect and brings about an aberrant synchronization in neural activity (Chaves and Sander, 2005; Thomas et al., 2006). Based on the *in vitro* study on mice in the neocortex, impairment in glutamate release, which is either due to the decreased function of glutamate receptors or due to loss of glutamate receptors, causes the generalized absence and tonic–clonic epileptic seizures (Seal et al., 2008). A study on the neocortex of mice (Maheshwari et al., 2013) has shown that the paradoxical seizure exacerbation effect of the antiepileptic medication can be explained by the unintended suppression of inhibitory interneurons following the NMDA receptor blockade. Additionally, in an *in vivo* study on a mouse model of tuberous sclerosis complex (TSC), the mutation in either TSC1 or TSC2 can disturb the function of NMDA receptors in the EI, which in turn can cause a shift from normal activity to ictal activity (Lozovaya et al., 2014). However, the mechanisms underlying the transition from interictal to ictal activities associated with glutamatergic receptors on EI remained unclear. Here, we take c_py–ei_ as the growing bifurcation parameter to investigate the dynamics of a model to explore the epileptiform activity induced by the glutamatergic receptor function of EI. According to Figure 3, by gradually decreasing the EI excitability in the cortex, we can observe rich epileptiform transition dynamics.

According to the bifurcation diagram in Figure 3, from right to left, we can observe the normal background activity, preictal spikes, clonic seizures, absence seizures, slow rhythmic activity, and tonic seizures, respectively, combined with corresponding time series, as the parameter c_py–ei_ decreases. Hence, suppression of glutamatergic receptors on EI can lead to the transition from interictal to preictal signals, and also transition between epileptic seizures. With decreasing synaptic strength c_py–ei_, the system encounters normal background activity (Figure 3A) for the normal value of c_py–ei_. As c_py–ei_ becomes smaller, a supercritical Hopf bifurcation point (H_1_) happens and the stable fixed points become unstable at c_py–ei_ ∼0.74. Upon approach of c_py–ei_ to ∼0.73, a period-doubling bifurcation (PD1) takes place and shapes the two-amplitude preictal spike pattern (Figure 3B). With further decrease of the c_py–ei_ parameter to ∼0.72, another period-doubling bifurcation (PD2) happens and then starts to form clonic seizure patterns (Figure 3C). In addition, with a further decrease in the c_py–ei_ parameter to ∼0.678, a bistable region including two stable limit cycles takes place between the first- and second-fold limit cycle bifurcations (LPC1 and LPC2) and shapes the SWD patterns (Figure 3D). As the c_py–ei_ parameter decreases to ∼0.591, the first subcritical Hopf bifurcation (H_2_) occurs and the stable points appear again. The second bistable region consists of stable focus points and a stable limit cycle happens between the subcritical Hopf bifurcation (H_2_) and the third fold limit cycle (LPC3) until c_py–ei_ is approaching to ∼0.532. Upon decreasing the c_py–ei_ parameter, slow rhythmic activity (Figure 3E) happens, and then at c_py–ei_ ∼0.451 the second supercritical Hopf bifurcation (H_3_) arises from the steady state and shapes the tonic seizure patterns (Figure 3F).

Altogether, this model showed that impairment of GABAergic or glutamatergic receptors in the EI population causes the transition from normal background activity to seizures (preictal signal) and the transition between absence, tonic, and clonic seizures. This model shows the possible existence of supercritical Hopf bifurcation with growing amplitude oscillations when the transition from interictal to ictal states occurs. In order to have a deeper understanding of the interictal to ictal transition dynamics, we investigate the bifurcation of the cortical and sensory input frequencies in our model.

#### Hybrid Cooperation of GABAergic and Glutamatergic Receptors of Excitatory Interneurons in Epileptic Transition Dynamics

**FIGURE 4:**
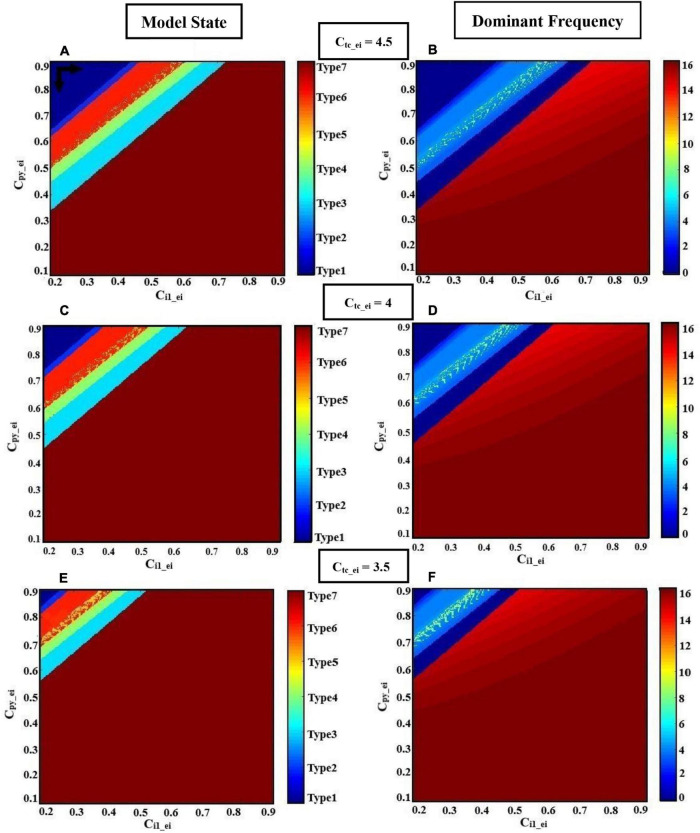
Hybrid modulation of the model. Patterns of evolutions of the model states are shown when the glutamatergic and GABAergic synaptic strength of EI (c_py–ei_, c_i1–ei_) in the cortex are changed. Each row corresponds to a given TC to EI excitatory synaptic strength (c_tc–ei_). The left column shows the states (normal or epileptic activities), and the right column shows the dominant frequencies (DF) of the model output. We set a_tc_ = 0 and a_py_ = 0. The various activities **(A,C,E)** and their corresponding dominant frequencies **(B,D,F)** with decreasing excitatory synaptic strength c_tc–ei_ from top to bottom, 4.5, 4, and 3.5, respectively, are shown. The model produces different firing states with variation of c_py–ei_ and c_i1–ei_. These states are normal background firing (Type1; DF = 0 Hz), preictal activity (Type2; DF < 3.5 Hz), slow rhythmic activity (Type3; DF = 0 Hz), typical absence seizure (Type4; 2 Hz < DF < 4 Hz), atypical absence seizure (Type5; DF > 4 Hz and DF < 2 Hz), clonic seizure (Type6; DF ≤ 7 Hz), and tonic seizure (Type7; DF ≥ 14 Hz). The black horizontal and vertical arrows represent the transition routes from the normal state to the pathological states.

The genesis of generalized seizures requires interdependencies in different thalamocortical connections. Studies on genetic rat models have shown that the increasing excitatory coupling strength from the thalamus to the cortex may facilitate the maintenance and propagation of absence seizures (Sitnikova et al., 2008; Lüttjohann and Pape, 2019). However, based on the paradoxical behavior of glutamatergic and GABAergic receptors discussed in sections “Transition Dynamics Produced by GABAergic Receptor-Mediated Inhibition in Excitatory Interneurons,” and ““Transition Dynamics Produced by Glutamatergic Receptor-Mediated Excitation in Excitatory Interneurons,” here, we explore the effect of decreased glutamatergic synaptic strength from the thalamus to the neocortex on seizure propagation using the extended model. In this section, we investigate the effect of a dual collaboration of intra-EI GABAergic and glutamatergic synaptic strength in the cortex on the transition between different kinds of activity, either from interictal to ictal signal or from absence seizure to tonic and clonic seizures. Moreover, we explore the influence of the TC to EI pathway dominated by glutamatergic receptors on the initiation and propagation of epileptic activities based on the altered ratio of the dual cooperation of intra-EI GABAergic and glutamatergic receptors in the cortex. According to Figure 4, the pattern evolutions for different values of c_tc–ei_ are obtained to investigate the state transition produced by this model and their corresponding dominant frequency distributions as the parameters of c_py–ei_ and c_i1–ei_ varies in region [0.1,0.9] × [0.2,0.9].

For higher values of synaptic strength like c_tc–ei_ = 4.5 (Figures 4A,B), this model demonstrates seven different types of activities, when ci1-ei varies from 0.2 to 0.9. We denoted these seven activities as follows: type1, normal background activity (DF = 0 Hz in Figure 4B); type2, preictal activity (DF < 3.5 Hz in Figure 4B); type6, clonic seizure (DF ≤ 7 Hz in Figure 4B); type5, atypical absence seizure (DF > 4 Hz and DF < 2 Hz in Figure 4B); type4, typical absence seizure (2 Hz < DF < 4 Hz in Figure 4B); type3, slow rhythmic activity (DF = 0 Hz in Figure 4B); type7, tonic seizure (DF ≥ 14 Hz in Figure 4B). Upon decreasing the synaptic strength c_tc–ei_ from 4.5 to 4 (Figures 4C,D), one can see that the surface of the yellow region (atypical absence seizure; Type5) and red region (tonic seizure; Type7) increases with the reduction level of excitation of intra-EI. Accordingly, the regions correspond to normal background activity (Type1), preictal activity (Type2), clonic seizure (Type6), typical absence seizure (Type4), and slow rhythmic activity (Type3) decreases. Biologically, decreasing the excitatory coupling strength from TC to EI interrupts the normal activity of the thalamic relay nucleus in the thalamocortical network. This interruption facilitates the transition from interictal to ictal, i.e., with the increasing of c_py–ei_ and c_i1–ei_ from their normal state, the transition from normal background activity to ictal activity and also the transition between clonic, absence, and tonic seizures can occur more easily. Upon further decreasing of c_tc–ei_ to 3.5 (Figures 4E,F), the yellow and red regions correspond to atypical absence seizure (Type5) and tonic seizure (Type7) extend, respectively. However, the whole other regions decrease, which shows that the transition from normal background activity to epileptic seizure activities in this model can be influenced by the intra-EI GABAergic and glutamatergic receptors in the cortex under the impact of the thalamus to the cortex synaptic strength.

In summary, the intra-EI GABAergic and glutamatergic receptors in the cortex can elicit seven different kinds of activity in this model under the effect of excitatory synaptic strength from TC to EI. The lower excitatory synaptic strength of c_tc–ei_, in the model causes a faster transition from interictal to ictal state. This implies the important role of the thalamus to cortex synaptic strength in the initiation and maintenance of generalized epileptic seizures.

#### Transition Dynamics Produced by Glutamatergic Receptor-Mediated Excitation in Reticular Nucleus

**FIGURE 5:**
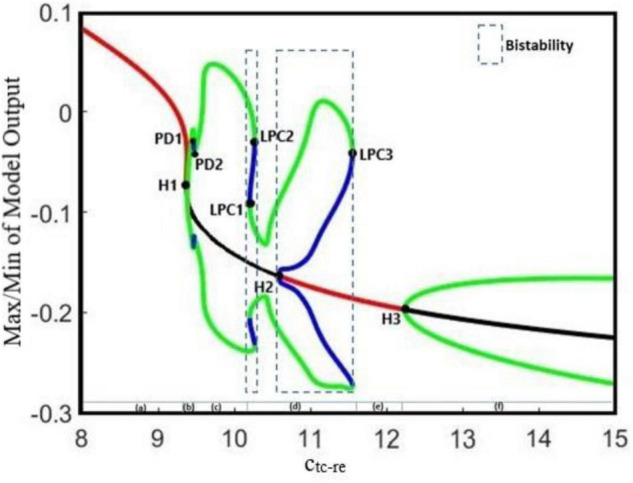
The bifurcation diagram for the bifurcation parameter c_tc–re_. Other parameters of the model are c_py–ei_ = 0.75, c_i1–ei_ = 0.33, and c_tc–ei_ = 4.2. We also set a_tc_ = 0 and a_py_ = 0. In the bifurcation diagram, blue and green cycles represent the unstable and stable limit cycles, respectively. The red and black lines represent the stable and unstable points, respectively. LPC1, LPC2, and LPC3 are fold limit cycle bifurcation, H1 and H3 are supercritical Hopf bifurcation, H2 is subcritical Hopf, and PD1 and PD2 are period doubling bifurcations.

Increasing the GABAergic inhibition is predominantly believed to suppress epileptic seizures; however, studies (Klaassen et al., 2006; Cope et al., 2009; Wong, 2010) have reported that enhanced GABAergic inhibition in the brain promotes seizure as well. The reticular nucleus (RE) is mainly composed of inhibitory interneurons that their epileptogenic role in seizure is investigated by Liu and Wang (2017) in their thalamocortical model. Liu and Wang (2017) did not consider the EI population in their model when they investigated the role of TC to RE synaptic strength in epileptic activities. Therefore, here, we investigate how the strength of the excitatory synapses of TC affects the function of the inhibitory reticular nucleus population in seizure generation in the presence of EI. By increasing the strength of the excitatory synapses from TC to RE, we increase the inhibitory behavior of RE in the proposed thalamocortical model. In Figure 5, we show that the proposed model demonstrates a rich dynamic and that the transition from interictal to ictal occurs when we increase c_tc–re_. In Figure 5, from left to right, first, we observe the normal background activity. After increasing the parameter c_tc–re_ to ∼9.4, the transition from steady state to the limit cycle of preictal (supercritical Hopf bifurcation H1) happens. Further increasing the c_tc–re_ to ∼9.58 and 9.63, the first and second period-doubling bifurcations (PD1, PD2) take place, respectively. After PD2, the limit cycles corresponding to clonic seizure take place. With increasing of c_tc–re_ to ∼10.1 the first fold limit cycle (LPC1) and then at c_tc–re_ ≈ 10.3 the second fold limit cycles (LPC2), the model generates the first bistable region between LPC1 and LPC2, which shape the limit cycle corresponding to absence seizure activity. The second bistable region occurs between the subcritical Hopf bifurcation H2 at ∼10.6 and the third fold limit cycle LPC3 at ∼11.9. Then, at c_tc–re_ ≈ 11.8 the transition from limit cycles of absence seizures and steady state occurs. Upon increasing of c_tc–re_ to ∼12.1, the transition from the stable equilibrium to the limit cycle of tonic seizure happens. By increasing the parameter c_tc–re_ to 9.5, we can observe the first supercritical Hopf bifurcation (H1) and the transition from interictal to preictal state. Then by further increasing c_tc–re_ and inducing more impairment in the TC to RE pathway, we can observe that limit cycles correspond to clonic and absence seizures, respectively.

### Frequency Analysis of the Different Initial States of the Model

According to clinical observations, sensory (Dawson, 1947) and cortical (Bezard et al., 1999) stimulations can provoke epileptic seizures. Therefore, in this section, we examine the effect of sensory and cortical inputs in the interictal to ictal transition using the extended thalamocortical model. Studies on thalamocortical models (Haghighi and Markazi, 2017, 2019) have shown that changes in the frequencies of cortical and sensory periodic inputs can cause a transition from chaotic to periodic activities in the output of the model. Therefore, we will first examine the effect of frequencies of the cortical and sensory inputs on the transition behavior of our model, and even more importantly, we will discuss the effect of the initial state of the model on this transition.

**FIGURE 6:**
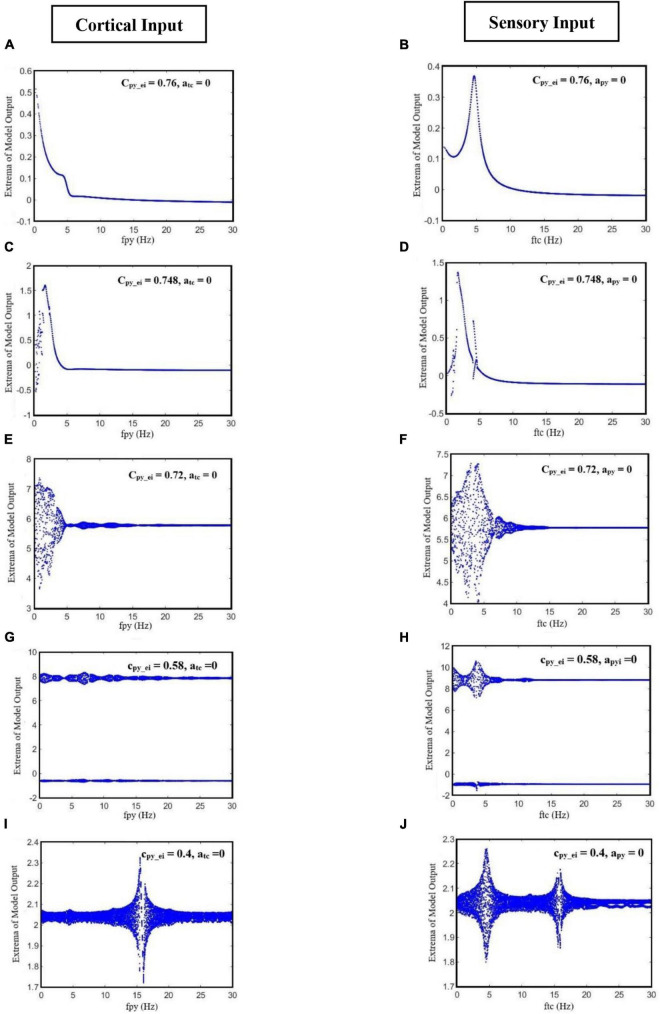
The bifurcation diagrams of the model output for different values of c_py– ei_, when the frequencies of the cortical input (fpy) and sensory input (ftc) change. Other parameters have the numerical values of c_i1–ei_ = 0.3 and c_tc–ei_ = 4.5. For the bifurcation diagrams shown in the left column **(A,C,E,G,I)**, fpy is the bifurcation parameter and a_tc_ has been set to zero; and for the bifurcation diagrams of the right column **(B,D,F,H,J)**, ftc is the bifurcation parameter and a_py_ is equal to zero.

Figure 6 shows various bifurcation diagrams for different initial states of the model, such as normal background activity (interictal), preictal activity, and clonic, absence, and tonic seizures. To change the initial state of the model, we used different values for parameter c_py_ei_. These values were selected using the results of the bifurcation analysis shown in Figure 3, in which c_py_ei_ is the bifurcation parameter. In Figures 6A,B, we set c_py_ei_ = 0.76, which is in the range corresponding to interictal activities. In Figures 6C,D, by decreasing the parameter c_py_ei_ to 0.748, the initial state of the model corresponds to preictal activities. In Figures 6E,F, we change the initial state of the model to a clonic seizure state by decreasing the value of c_py_ei_ to 0.72. As it is shown in Figures 6G–J, by further decreasing c_py_ei_ to 0.58 and then to 0.4, we set the model in absence seizure and then in tonic seizure states, respectively.

On the other hand, for each state of the model, we changed the frequencies of cortical and sensory periodic inputs separately with a frequency step increment of 0.01 Hz. For the interictal initial state (Figures 6A,B), the model shows a linear resonant behavior with a resonant frequency of fpy ≈ 0.2 and ftc ≈ 4.7 as the cortical and sensory input frequencies increase. The model, in this case, behaves like a bandpass filter for the sensory input and a low pass filter for the cortical input. For c_py_ei_ = 0.748, when the frequency of cortical input is increased, a chaotic behavior is resulted. In Figure 6C, with further increase in the cortical input frequency (fpy), we notice a jump near the frequency of fpy ≈ 1.7. Moreover, by increasing the frequency of the sensory input, two jumps take place near the frequencies of ftc ≈ 1.8 and ftc ≈ 4 (Figure 6D). Then, upon increasing fpy and ftc, the model returns to its normal periodic behavior. This observation indicates that, when the initial state of the model changes from normal background activity to preictal activity, the behavior of the model changes from a linear resonator to a non-linear resonator. In Figures 6E,F, where the model is in the clonic seizure state, by increasing the frequencies of cortical and sensory inputs, we observe that the model first displays a noticeable chaotic behavior around 3 Hz, which is the main frequency of the clonic seizure, and then returns to its expected periodic behavior. In Figures 6G,H, when the model is in the absence seizure state, increasing the cortical and sensory input frequencies yields two chaotic behaviors with two different amplitude ranges; the reason for this is the coexistence of the two different SWD patterns with different amplitudes. This chaotic behavior can be seen around the main frequency of absence seizure (fpy ∈ [2, 4]). As we can see in Figures 6I,J, when the state of the model changes to tonic seizure activities, we observe a chaotic behavior around the main frequency of tonic seizure (fpy ≈ 16). As it is demonstrated in Figures 6F,H,J, when the model is in the seizure activity state, by increasing the frequency of sensory input, two peaks in the frequency response of the model are observed. These two peaks can be seen more clearly in tonic seizures (Figure 6J) and correspond to the two jumps already observed in Figure 6D.

**FIGURE 7:**
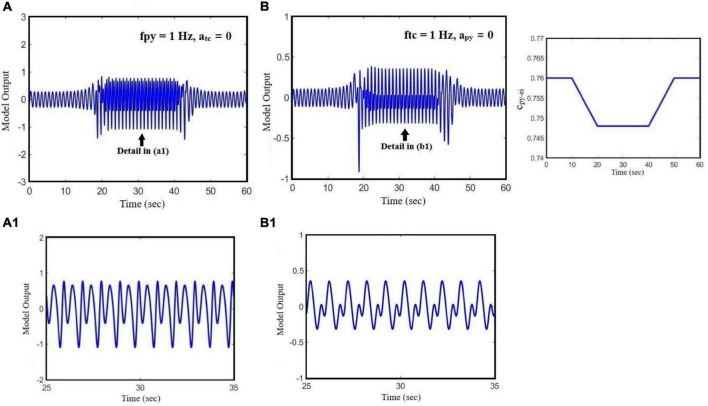
The effect of state transition of the model on its output when the parameter of c_py_ei_ changes. The effect of the initial state of the model on the transition between periodic signal and seizure when the cortical and sensory input frequencies are constant. In **(A)**, we set the parameters of fpy = 1 Hz and atc = 0. In **(B)**, we set ftc = 1 HZ and a_py_ = 0. The pattern of glutamatergic synaptic strength, c_py_ei_, is presented on the right-hand side diagram.

Figure 7 demonstrates the effect of the initial state of the model on the transition between small-amplitude oscillation and large-amplitude seizure activities, all in the time domain. We set c_py_ei_ equal to 0.76 in order to adjust the model in its normal state, and then we change the model state from interictal (normal activity) to preictal activity by decreasing the c_py_ei_ parameter to 0.748. In Figure 7A, the model receives a cortical input with a fixed frequency, fpy = 1 Hz, in the absence of sensory input. However, in Figure 7B, we only consider the effect of the sensory input on the output of the model and we set its frequency ftc = 1 Hz. In Figures 7A,B, it is shown that based on the bifurcation diagram of Figure 3, by changing c_py_ei_ from 0.76 to 0.748, the state transition from normal activity to typical absence seizure activity occurs, and then it returns to the normal activity as c_py_ei_ returns to its initial value.

## Discussion

**FIGURE 8:**
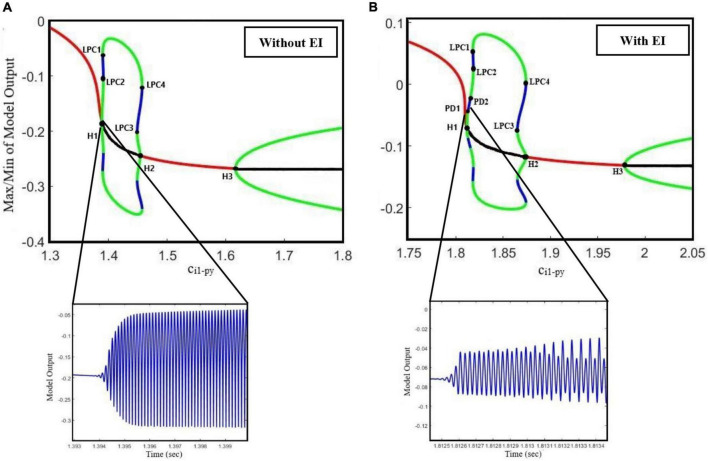
**(A)** Bifurcation diagram of the output of the model when the excitatory interneurons are not considered in the model (parameters are set based on the caption of Figure 1). The bifurcation parameter is the inhibition strength between I1 (fast interneurons) and PY (pyramidal) population (c_i1_py_). **(B)** Bifurcation diagram of the output of the model when the excitatory interneurons are considered in the model (c_py_ei_ = 0. 8, c_i1–ei_ = 0.3, c_tc–ei_ = 4.5). The bifurcation parameter is the inhibition strength between I1 (fast interneurons) and PY (pyramidal) population (c_i1_py_).

Traditionally, mechanisms underlying seizures have been considered to be due to increased excitation, decreased inhibition, or even both of them resulting in hyper-excitability and seizure generation. However, glutamatergic and GABAergic neurotransmitters can exert paradoxical effects and cause seizures. Antiepileptic drugs do not necessarily work by decreasing excitation and increasing the inhibition of neural activities. Studies Cossart et al. (2005), Fritschy (2008), Kaila et al. (2014), Knoflach et al. (2016), and Trevelyan (2016) have shown that some seizures occur when inhibition is enhanced in the brain. Therefore, it might be oversimplifying if one considers the role of GABAergic inhibition in the brain as the only antiepileptics. In fact, understanding the seemingly contradictory role of the excitatory and inhibitory neurons in the brain can lead to new therapies for epileptic seizures. Therefore, in the present study, we investigated the opposite effect of GABAergic and glutamatergic receptors in seizure onset through the dynamical analysis of an extended model. In this direction, we proposed an extended neural mass model, which considers the role of the spiny stellate cell population connectivity with other cortical neural populations in different states of seizure generation. The original thalamocortical model, which was developed by Liu and Wang (2017), was modified and extended and then used to simulate the preictal activity that has a prevailing role in epileptic seizure prediction. Using this model, we investigated the interactions between EI and the glutamatergic pyramidal and inhibitory interneurons in the cortex, and how they lead to epileptic seizures. This model enabled us to generate preictal activities before the clonic seizure. To make this point clearer, we calculated the bifurcation diagram of the output of the model for **c_*i1*–*py*_**, as the bifurcation parameter, in two cases: (1) without the EI population in the model and (2) at the presence of EI. As one can see in Figures 8A,B by increasing the inhibitory synaptic strength between PY and I1, first a clonic seizure and then a tonic seizure occur. In Figure 8A, when the EI population is not considered in the model, there exist no preictal activities (please notice the high-amplitude clonic activity after Hopf bifurcation H1). However, in Figure 8B, when the role of EI in the model is considered, the preictal activity emerges in the bifurcation diagram right after Hopf bifurcation H1 (please notice the low-amplitude preictal activity after H1). Therefore, one can conclude that although the order and list of bifurcations that occur when the inhibition between fast interneurons and pyramidal neurons increases are the same for both cases (extended model and the original model developed by Liu and Wang, 2017), the emergence of the preictal activities in the model depends on the EI. Without these neurons (and certainly the feedback loop created by them), the model does not show any preictal activities and the seizure starts abruptly.

Our results show the richness of the dynamics that the proposed model can generate, including normal background activity, preictal spikes, slow rhythmic activity, clonic seizure, typical absence seizure, and tonic seizure, as we decreased and increased the glutamatergic and GABAergic synaptic strengths of the EI, respectively. Furthermore, bifurcation diagrams of the model were obtained by varying the coupling strength of GABAergic and glutamatergic receptors in EI. The diagrams in Figures 2, 3, 5 show that the interictal to ictal transition occurs when we increased the glutamatergic excitation and GABAergic inhibition in the cortex. Based on our bifurcation diagrams, pathological transitions are consequences of supercritical and subcritical Hopf bifurcations as well as the fold limit cycle bifurcation. The onset of preictal discharges is characterized by a supercritical Hopf bifurcation. This results in a preictal activity, which is characterized by growths in amplitude and frequency of neural activities. As mentioned before, Liu and Wang (2017) developed a thalamocortical model, which is originally inspired by Taylor et al. (2014) and Fan et al. (2015) to study the epileptogenic role of the synaptic strength between TC and ER in the thalamus. Using that model, they did not consider the spiny stellate cell population and its role in epileptic seizure generation. One of the most important features lacking in their results was the preictal state of the model. In fact, the model was not able to generate the limit cycles of preictal state in their bifurcation analysis when they investigated the excitatory pathway from TC to RE. As a result, in their bifurcation diagrams there was an abrupt appearance of ictal limit cycles after the normal background activities. However, in Figure 5 we evaluated the extended thalamocortical model in a more general framework and we showed that this model is capable of generating the preictal state when the synaptic strength from TC to RE is changed. We showed that considering the role of EI was crucial for the emergence of the preictal state. Using this model, we demonstrated that ictal discharges do not appear abruptly after a period of interictal activities. As it was shown in the bifurcation diagrams (Figures 2, 3, 5), we can simulate the gradual increase in frequency and amplitude from normal activities to ictal activities. Consequently, the model demonstrates the key role of the spiny stellate cells of the cortex in interictal to ictal transition dynamics.

In order to have a deeper insight into the dynamics of interictal to ictal transition, we also explored the effect of cortical and sensory periodic (sinusoidal) inputs on the output of the extended and modified model. Our simulation results reveal that the transition from normal activity to seizure activity occurs when a perturbation in the dynamic structure of the model relocates the model state from interictal to preictal state. Our results show that during the preictal state, the extended model is more susceptible to sensory and cortical inputs, which in turn causes typical absence seizures. Moreover, the frequency response of the model (Figure 6) makes it clear that model responses to the cortical and sensory input are dependent on the initial state of the model. According to the bifurcation analysis depicted in Figure 6, when the model is in its normal state, it behaves as a linear resonator. Therefore, when we stimulate the model with sensory and cortical stimuli, the model output does not show a chaotic behavior and comes back to its normal behavior as the input cortical and sensory frequencies are increased. On the other hand, when any impairments occur in the glutamatergic receptors of the EI, these cause the model to enter the preictal state. Then, the same sensory and cortical stimulations can cause the model to act as a non-linear resonator and we can observe chaotic behaviors and jump phenomenon. This non-linear behavior increases when the initial state of the model changes to the ictal state. In general, the cortical stimulations evoke more peak-to-peak amplitudes of cortical output in this model, in comparison to sensory stimulations, when it is in clonic and tonic seizure states. On the contrary, the peak-to-peak amplitude of the model output when it is in the seizure activity state is more intense when it receives sensory stimulations. This demonstrates that absence seizures are more sensitive to sensory stimuli than cortical stimulations. Also, we investigated the effect of the initial state of the model in absence seizure generation. As is shown in Figure 7, when the extended model is in its normal state, we observe the low-amplitude activities, which correspond to normal background activity. However, by changing the model state from normal to preictal state, we can observe that the absence seizures take place. According to our observation, we suggest that alteration in the initial states of the brain can be considered as one of the principal causes of the absence and photosensitive seizures.

Finally, we examined the role of the thalamus in epileptic seizure activities. We investigated the role of cooperation of glutamatergic and GABAergic receptors of EI under the effect of the thalamic relay nucleus (TC) in seizure generation and propagation. As is shown in Figure 4, the results have shown the dependence of cortical function on the thalamic one in the initiation, propagation, and termination of epileptic seizures. Recent studies Chang et al. (2020) and Prathaban and Balasubramanian (2020) did not consider the thalamus and its synaptic connectivity with other neuronal populations such as EI in the cortical models they worked with. However, based on the bifurcation analysis obtained from the hybrid modulation of intra-EI excitatory and inhibitory synaptic strength in the extended neural mass model, the pathway from the thalamic relay nucleus to the EI facilitates the transitions between different types of epileptic activities, either from interictal to ictal or between clonic, absence, and tonic seizures. We believe that these results provide useful insights for understanding more thoroughly the function of the EI population and dynamics caused by their connections to other populations in the thalamocortical circuitry and the transitions between interictal to preictal and ictal states. Therefore, the extended model is more suitable to be used in epileptic seizure prediction and abatement.

## Data Availability Statement

Publicly available datasets were analyzed in this study. This data can be found in the following address: https://github.com/SabaTabatabaee/Neural-Mass-model-for-Epilepsy.

## Author Contributions

ST, FB, and MJ guaranteed the integrity of the entire study. ST contributed to literature research, formal analysis, and writing the original draft. FB and MJ contributed to project administration and reviewed and edited the manuscript. All authors contributed to conceptual design of the study, methodology, and validation.

## Conflict of Interest

The authors declare that the research was conducted in the absence of any commercial or financial relationships that could be construed as a potential conflict of interest.

## Publisher’s Note

All claims expressed in this article are solely those of the authors and do not necessarily represent those of their affiliated organizations, or those of the publisher, the editors and the reviewers. Any product that may be evaluated in this article, or claim that may be made by its manufacturer, is not guaranteed or endorsed by the publisher.
